# Dynamical modeling and analysis of the impact of zonal prevention and control under normalized management on African Swine Fever transmission in China

**DOI:** 10.1016/j.idm.2026.03.011

**Published:** 2026-03-25

**Authors:** Juan Li, Junhui Zhang, Lu Gao, Shubo Li, Huaiping Zhu

**Affiliations:** a School of Computer Science and Technology (School of Artificial Intelligence), Zhejiang Sci-Tech University, Hangzhou, PR China; b China Animal Health and Epidemiology Center, Qingdao, PR China; c Liaoning Center for Animal Disease Control and Prevention, Shenyang, PR China; d School of Animal Science and Technology (School of Animal Medicine), Huazhong Agricultural University, Wuhan, PR China; e LAMPS and Centre of Diseases Modeling CDM, Department of Mathematics and Statistics, York University, Toronto, M3J 1P3, ON, Canada

**Keywords:** African swine fever, Zone-based control, Dynamic transmission model, Deep neural network, Time-varying reproduction number

## Abstract

**Background:**

China's first outbreak of African swine fever (ASF) occurred in August 2018, rapidly spreading to all 31 provinces within three months, causing massive losses to the pork industry. After emergency measures such as a nationwide ban on swill feeding and enhanced biosecurity halted the initial epidemic wave, management transitioned to routine zone-based control. However, regional disparities in farm density and inconsistent policy implementation still pose challenges to effective prevention. Static-parameter models commonly used in ASF research fail to capture the dynamic nature of viral spread, and quantitative assessments of zone-specific interventions remain limited. Based on China's field experience, this study investigates how regional heterogeneity drives ASF transmission and quantifies the effectiveness of zonal control strategies, providing an evidence-based foundation for optimizing interventions.

**Methods:**

Monthly records of ASF-related deaths and culls from August 2018 to December 2019 were collected to construct a three-dimensional non-autonomous transmission model (swine-swill-environment) with time-varying parameters. A fully connected feed-forward neural network (FNN) was trained using PyTorch to approximate these parameters. Optuna was employed to optimize the network architecture, and a composite loss function was defined for joint parameter estimation. The effective reproduction number was computed in real-time to quantify transmission risk. Global sensitivity analysis was performed using Latin-hypercube sampling combined with partial rank correlation coefficients (PRCC) to evaluate the impact of zone-specific policies and single or combined interventions (swill-feeding ban, biosecurity compliance, safe carcass disposal).

**Results:**

Model-based scenario simulations indicate that zone-based control significantly suppressed ASF transmission, with effectiveness influenced by implementation timing and regional balance. Relative to no zoning, initiating zones in September 2018 reduced peak infected pig numbers by 26% and total deaths by 63%. When zoning began in October with a balanced (1:1) farm-density ratio, overall mortality decreased by 30% compared with an unbalanced (4:1) ratio, confirming "early plus balanced zoning" as the optimal strategy. Among single interventions evaluated against an October-initiated 2:1 farm-density baseline, doubling biosecurity awareness reduced total deaths by 62%, whereas intensified safe carcass disposal was positively correlated with mortality. A standalone swill-feeding ban was only marginally effective compared to zoning alone. However, combining all three interventions significantly reduced cumulative deaths to close to 480 thousand heads, demonstrating a clear synergistic effect.

**Conclusion:**

Swill feeding and pronounced regional heterogeneity are critical drivers of ASF spread. Combined interventions consistently outperform single measures. An early, balanced zoning strategy coupled with heightened producer biosecurity awareness represents the most effective management approach. These findings provide robust evidence to refine ASF prevention policies in China and serve as a technical reference for global epidemic management.

## Graphical abstract

**Figure:**
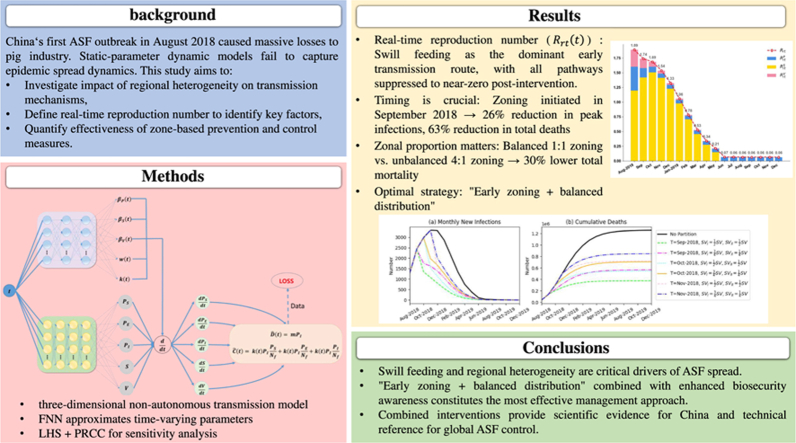

## 1 Introduction

African swine fever (ASF), one of the most devastating swine diseases worldwide, was first reported in Kenya in 1921 (Costard et al., 2009). According to the 72nd report by the World Organization for Animal Health (WOAH) issued in January 2026, ASF outbreaks were recorded in 71 countries across four continents between January 2022 and December 2025. During this period, the epidemic infected more than 1.137 million domestic pigs and 43,506 wild boars, causing approximately 2.32 million domestic pig deaths or culls (AFRICAN SWINE FEVER (ASF) Situation Report 72, 2026). Europe, with 5293 outbreaks among domestic pigs and 27,302 in wild boars, experienced the most severe impact, losing more than 1.62 million domestic pigs. Asia and Africa reported 7480 and 1128 domestic pig outbreaks, respectively, highlighting ASF risks in regions of high farming density. Approximately 26% of outbreaks occurred in areas with domestic pig densities exceeding 10 heads per square kilometer (AFRICAN SWINE FEVER (ASF) Situation Report 72, 2026).

ASF is a rapidly spreading viral infection affecting pigs of all breeds and ages, often resulting in mortality rates approaching 100% (African Swine Fever Prevention and Control Understanding Paper, 2018; Alonso et al., 2018). Transmission occurs through direct contact, contaminated fomites, arthropod vectors, and wild boar movements. This complexity in ASF epidemiology severely limits the effectiveness of traditional control methods (Kalenzi Atuhaire et al., 2013; Korennoy et al., 2017). Crucially, the ASF virus has a complex genome, and no effective vaccine or antiviral treatment has yet been developed. Consequently, biosecurity measures and stamping-out procedures remain the only available control strategies (Chenais et al., 2019; African Swine Fever Prevention and Control Understanding Paper, 2018). The spread of the virus is further complicated in developing countries by high farm density and inadequate veterinary infrastructure. In China, the initial ASF outbreak in August 2018 spread to all 31 provinces within three months due to long-distance pig transportation, swill feeding, and mechanical transmission by infected personnel and vehicles (X. Li & Tian, 2018; Qiu HJ, 2018; Wang QH et al., 2018). Early data indicated approximately 40% of outbreaks were directly linked to swill feeding (Yang HL et al., 2020), creating a local transmission cycle of "swill introduction–slaughterhouse spread–environmental persistence". In response, China implemented a nationwide ban on feeding kitchen waste to pigs in October 2018. This measure significantly reduced swill-associated cases from 40% to zero by June 2019 (J. Li et al., 2024; Notice of the General Office of the State Council on Further Improving the Prevention and Control of African Swine Fever, 2018), demonstrating the effectiveness of targeted interventions.

As the epidemic entered a chronic prevention phase, China progressively adopted a zone-based control system to mitigate the long-term threat of ASF. In June 2019, the Ministry of Agriculture and Rural Affairs (MARA) initiated regionalized prevention and control, selecting the six-province South-Central zone as a pilot region. The joint command for major animal-disease control in these provinces subsequently issued the *Regional Prevention and Control Plan for African Swine Fever and Other Major Animal Diseases in Central-Southern China* (Regionalized Prevention and Control Plan for Major Animal Diseases Such as African Swine Fever in Central and Southern China, 2019). In April 2021, MARA released the *Trial Zoned Prevention and Control Plan for African Swine Fever and Other Major Animal Diseases* (Notice of the Ministry of Agriculture and Rural Affairs on the issuance of the "Work Plan for the Prevention and Control of Major Animal Diseases such as African Swine Fever by Zoning (Trial)", 2021), dividing the country into five large zones: North, East, South-Central, Southwest, and Northwest. Differentiated control measures are adopted across these zones, including culling, active surveillance, enhanced biosecurity, and standardized disposal of pigs. Pig transportation is strictly regulated, with restrictions on inter-zonal movement and supervised point-to-point transport between zones. By mitigating cross-regional virus transmission, this policy provides a practical regulatory context for the model establishment and interpretation of conclusions in this study. Guided by principles of "epidemic prevention priority, zoned promotion, joint prevention and control, risk reduction, scientific prevention and control, and supply assurance", the plan mandates zonal management of ASF and other major animal epidemics (Wang Xu, 2021). Nevertheless, current zonal control systems face emerging challenges. Significant within-zone heterogeneity in farm density and uneven implementation of policies hinder uniform risk mitigation. Therefore, quantitatively evaluating the effectiveness of zonal strategies, considering interactive transmission among pigs, swill, and the environment, as well as producer behavior, has become essential for optimizing ASF prevention and control policies.

Existing dynamic models are essential tools for elucidating ASF transmission patterns and evaluating control measures. Classical Susceptible–Infectious–Removed (SIR) and Susceptible-Exposed-Infectious-Removed (SEIR) frameworks, along with variants such as Susceptible-Infectious-Survivor-Infected carcass (SICD) and Susceptible-Exposed-Infectious-Dead (SEID), have simulated outbreak trajectories under different intervention scenarios, highlighting the effectiveness of stamping-out, environmental disinfection, and swill-feeding bans. For instance, O'Neill et al. (O'Neill et al., 2020) developed an SICD model to compare ASF incidence under alternative policies, concluding that integrated strategies are more effective in disease elimination. Zhang et al. (X. Zhang et al., 2021) used an SEID model to examine ASF dynamics within commercial herds, demonstrating that compulsory culling of all pigs is unnecessary for containment. Barongo et al. (Barongo et al., 2016) employed a stochastic dynamic model to quantify how control timing influences disease mortality. Kouidere et al. (Kouidere et al., 2021) formulated an SIR framework addressing ASF transmission between pigs and ticks, concluding that protecting susceptible pigs is the most cost-effective measure. Lim et al. (Lim et al., 2024) applied an extended SEIR model to simulate wild-boar mortality in Singapore, emphasizing that restricting infected pig movement and blocking fomite-mediated spread are critical control elements. Song et al. (Song et al., 2022) incorporated environmental virus and swill transmission into an ASF dynamic model, showing both factors sustain ASF persistence. Li et al. (Juan Li et al., 2022; J. Li et al., 2024) simulated regional ASF spread and demonstrated that discontinuing swill feeding effectively reduced transmission. They developed a model explicitly incorporating swill and environmental contamination to quantify the impact of China's nationwide swill-feeding ban on ASF dynamics. However, most existing models employ static parameters, failing to account for variations in farm density, dynamic policy adjustments, and producer behavioral responses. Recently, integrating deep-learning techniques into dynamic transmission modeling has emerged as a promising approach to overcoming these limitations. For example, He et al. (He et al., 2023) combined data-driven deep learning with mechanistic modeling to propose a transmission dynamics-informed neural network (TDINN), quantifying multi-region COVID-19 intervention intensities. Zhang et al. (Z. Zhang et al., 2024) developed a hybrid model coupling dynamic modeling with deep neural networks to study brucellosis transmission, using time-varying parameters to evaluate evolving intervention strengths.

Currently, quantitative analyses of zonal ASF control remain limited, and no transmission models have integrated swill-mediated spread explicitly with spatial heterogeneity. Motivated by China's ASF prevention practices, this study proposes a hybrid dynamical model combining mechanistic and data-driven methods. The model is calibrated dynamically by a deep neural network using reported ASF mortalities and stamping-out records. This approach enables quantitative assessment of how zonal intervention intensity shapes national transmission patterns, providing evidence-based insights to optimize regional management strategies while balancing disease containment and production continuity.

## 2 Materials and methods

### 2.1 Data sources

The China Animal Health and Epidemiology Center (CAHEC), in collaboration with local animal disease control agencies, conducted detailed epidemiological investigations on every reported ASF outbreak (Gao et al., 2021). Upon completion, thorough investigation reports were submitted to the Ministry of Agriculture and Rural Affairs (MARA). These reports included comprehensive details on outbreak response measures and affected premises. Based on these epidemiological reports, we assembled a database containing report date, precise geographic location, probable infection source, farm size, and outbreak-related cases and deaths.

The study compiled data on Chinese ASF outbreaks from August 2018 to December 2019, focusing on monthly counts of newly documented pig deaths and culls. To mitigate the impact of reporting delays and random fluctuations, we applied a three-month moving-average filter to smooth the data series (Wu et al., 2020). Specifically, each monthly value was replaced by the average of the current month and the two preceding months to minimize late-reporting bias. The smoothed series was then utilized for calibration and estimation of unknown model parameters.

Additionally, referencing previous literature (Juan Li et al., 2022; J. Li et al., 2024) and the *China Animal Husbandry and Veterinary Yearbook* (Editorial Board of China Animal Husbandry and Veterinary Yearbook, 2020), we obtained benchmark values for critical quantities. These included the monthly pig culling rate, farm-level disinfection coverage, environmental virus decay rate, number of ASFV-positive farms, transition rate from latent to infectious pigs, swill consumption rate, disease-related mortality, total number of pig farms in China, average number of farms subjected to sanitary culling after detection, and initial values for all state variables. These data were employed to quantify selected model parameters.

### 2.2 Transmission dynamical model

**Fig. 1:**
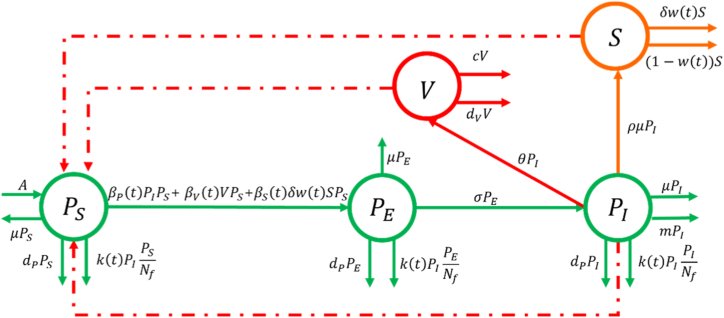
Transmission diagram of ASFV. The dashed and solid lines represent infection actions and the flow direction of research objects, respectively.

Consistent with the documented transmission routes of ASFV in China, susceptible pigs can become infected through three mechanisms: direct contact with infectious animals, contact with virus-contaminated environments, and ingestion of contaminated swill (Juan Li et al., 2022). Accordingly, the model emphasizes three core transmission compartments: pigs (*P*), contaminated swill (*S*), and contaminated environment (*V*). The pig population is further subdivided into three epidemiological states: susceptible ($P_{S}$), latent ($P_{E}$), and infectious ($P_{I}$). In our previous work (J. Li et al., 2024), we developed an autonomous live-pig-swill-environment interaction model to quantify how early-phase control measures, specifically the nationwide ban on swill feeding and compulsory sanitary culling, affected the initial trajectory of the Chinese ASF epidemic. However, that framework did not incorporate spatial-temporal heterogeneity or zone-based control effects, thus limiting its scope. Building on our earlier studies (Juan Li et al., 2022; J. Li et al., 2024), we now extend the model by embedding time-varying parameters to reflect both dynamically adjusted interventions and spatio-temporal variability in viral spread. This approach enables more accurate evaluations of how zonal strategies influence ASF dynamics under various scenarios. Specifically, we treat the following key quantities as explicit functions of time: the effective transmission rate from infectious pigs to susceptible pigs ($\beta_{P}\left(t\right)$), the effective transmission rate from contaminated swill to susceptible pigs ($\beta_{S}\left(t\right)$), the effective transmission rate from environmental ASFV to susceptible pigs ($\beta_{V}\left(t\right)$), the proportion of total swill used for pig feeding (w(t)), and the average number of farms subjected to sanitary culling after detection (k(t)). On this basis, we plot a schematic diagram depicting the dynamical transmission of ASFV between different compartments (Fig. 1) and formulate a non-autonomous dynamical system (Model (1)); the variables and parameters are detailed in Table 1, Table 2.

(1) $$\left\{ \begin{array}{c} \frac{dP_{S}}{dt}=A-\beta_{P}\left(t\right)P_{I}P_{S}-\beta_{V}\left(t\right)VP_{S}-\beta_{S}\left(t\right)\delta w\left(t\right)SP_{S}-d_{P}P_{S}-\mu P_{S}-k\left(t\right)P_{I}\frac{P_{S}}{N_{f}}\text{,}\\ \frac{dP_{E}}{dt}=\beta_{P}\left(t\right)P_{I}P_{S}+\beta_{V}\left(t\right)VP_{S}+\beta_{S}\left(t\right)\delta w\left(t\right)SP_{S}-d_{P}P_{E}-\mu P_{E}-k\left(t\right)P_{I}\frac{P_{E}}{N_{f}}-\sigma P_{E}\text{,}\\ \frac{dP_{I}}{dt}=\sigma P_{E}-d_{P}P_{I}-\mu P_{I}-k\left(t\right)P_{I}\frac{P_{I}}{N_{f}}-mP_{I}\text{,}\\ \frac{dS}{dt}=\rho \mu P_{I}-\delta w\left(t\right)S-\left(1-w\left(t\right)\right)S\text{,}\\ \frac{dV}{dt}=\theta P_{I}-cV-d_{V}V \end{array}\right.$$

**Table 1:** Description of state variables and parameters in the model (1).

State variables	Descriptions	Units	Values	References
$P_{S}\left(t\right)$	Number of susceptible pigs at time t	head	$P_{S}\left(0\right)=$ 3.1035e+08	Data
$P_{E}\left(t\right)$	Number of exposed pigs at time t	head	$P_{E}\left(0\right)=$ 426	Ref (J. Li et al., 2024)
$P_{I}\left(t\right)$	Number of infectious pigs at time t	head	$P_{I}\left(0\right)=$ 1567	Ref (J. Li et al., 2024)
S(t)	Weight of contaminated swills at time t	kg	*S*(0)= 68646	Ref (J. Li et al., 2024)
V(t)	Virus load in the environment at time t	TCID50	V(0)= 4.9968e+08	Ref (J. Li et al., 2024)

**Table 2:** Description of parameters in the model (1).

Fixed Parameters	Descriptions	Units	Values	References
A^a^	Recruitment rate of pigs	Head/month	2.621e+07 × 2.4× 10/12	Data
$\beta_{P}$	Effective infection rate among infectious pig to pig	1/(head. Month)	Fig. 3	Estimated
$\beta_{S}$	Effective infection rate of pig due to feeding containment swills	1/(head. Month)	Fig. 3	Estimated
$\beta_{V}$	Effective infection rate of pig contacting virus in the environment	1/(head. Month)	Fig. 3	Estimated
$d_{P}$^b^	Removed rate of pigs due to natural elimination or other diseases.	1/month	1/6.5 × 4%	Ref (Juan Li et al., 2022)
μ^b^	Offtake rate of pigs	1/month	1/6.5 × 96%	Ref (Juan Li et al., 2022)
k	Average number of farms that have been harmlessly treated		Fig. 3	Estimated
$N_{f}$	Total number of farms		4.6559e+07	Ref (Song et al., 2023)
σ^c^	Transfer rate from exposed pig to infectious pig	1/month	1/10 × 30	Ref (Juan Li et al., 2022)
m	Disease-related death rate for infectious pig	1/month	0.3734	Ref (J. Li et al., 2024)
θ^c^	Discharging quantity of ASFV by infectious pig in the farm	TCID50/(head. month)	2e+04 × 30	Ref (Juan Li et al., 2022)
$d_{V}$^c^	Decaying rate of ASFV in the environment	1/month	1/30 × 30	Ref (Juan Li et al., 2022)
C^d^	Removal rate of ASFV due to cleaning and disinfection	1/month	1/7 × 30× 0.8	Ref (Juan Li et al., 2022)
ρ	Transformation ratio of infectious pigs to swill	kg/head	296.6709	Ref (J. Li et al., 2024)
δ^c^	Consumption rate of swills by pigs	1/month	1/2 × 30	Data
w	Proportion of swill for raising pigs in the total amount	none	Fig. 3	Estimated

Note.

a China's annual breeding sow inventory is approximately 26.21 million heads. Each sow produces 2.4 L per year, averaging 10 piglets per litter. Thus, the monthly piglet reproduction rate was calculated as (2.621e+07 × 2.4 × 10)/12.

b Pigs require approximately 6–7 months to mature from weaning to market weight; thus, the average maturation period was set at 6.5 months. Reported mortality rates for finishing pigs and sows range from 3.0 % to 5.9 %, leading us to adopt a mean background mortality rate of 4%. Consequently, the monthly mortality rate due to natural causes or diseases other than ASF was calculated as 1/6.5 × 4%. The monthly slaughter/outflow rate was estimated to be 1/6.5 ≈ 15.4%.

c Parameters reported in reference (Juan Li et al., 2022) are expressed daily. To ensure consistency with our monthly time step, each rate was multiplied by 30.

d Reference (Yang HL et al., 2020) indicates that disinfection occurs at least once per week, and reference (Juan Li et al., 2022) reports a disinfection efficacy of 0.8 per event. Assuming four disinfection events per month, the monthly ASFV removal rate due to cleaning and disinfection was thus calculated as 1/7 × 30 × 0.8.

### 2.3 The real-time reproduction number

Model (1) is an extension of the autonomous model proposed in our previous study (J. Li et al., 2024). In the case where all time-varying parameters are constant, the model reduces exactly to the autonomous system analyzed therein, whose basic reproduction number $R_{0}$ is given by:

$$R_{0}=\left(\beta_{P}+\frac{\beta_{S}\delta w\rho \mu }{\delta w+\left(1-w\right)}+\frac{\beta_{V}\theta }{c+d_{V}}\right)\frac{P_{S}\left(0\right)\sigma }{\left(d_{p}+\mu +\sigma \right)\left(d_{p}+\mu +m\right)}\text{.}$$

However, the conventional $R_{0}$ cannot quantify the real-time transmission risk imposed on susceptible pigs by infected swine herds, contaminated swill, and environmental ASFV. To address this limitation, this study introduces the real-time reproduction number $R_{rt}\left(t\right)$*.* This metric incorporates time-varying factors and serves as a critical tool for measuring dynamic epidemic transmission risks. Based on the three ASFV transmission pathways and the constructed model, we define the real-time reproduction numbers for the respective pathways as follows: $R_{rt}^{P}$ represents the average number of secondary infections produced by one infectious pig during its infectious period. $R_{rt}^{S}$ represents the average number of secondary infections produced by 1 kg of contaminated swill during its circulation period; and $R_{rt}^{V}$ represents the average number of secondary infections produced by one unit of environmental ASFV during its survival period (Xue et al., 2018).

The instantaneous reproduction numbers $R_{rt}^{P}\left(t\right)$ ‘$R_{rt}^{S}\left(t\right)$ ‘$R_{rt}^{V}\left(t\right)$ are computed as follows:

(2) $$R_{rt}^{P}\left(t\right)=P_{P}\times \alpha_{P}\left(t\right)\times T_{P}\left(t\right)=\frac{\sigma }{d_{P}+\mu +\frac{k{\left(t\right)P}_{I}\left(t\right)}{N_{f}}+\sigma }\times \beta_{P}\left(t\right)P_{S}\left(t\right)\times \frac{1}{d_{P}+\mu +\frac{k{\left(t\right)P}_{I}\left(t\right)}{N_{f}}+m}\text{,}$$

(3) $$R_{rt}^{S}\left(t\right)=P_{S}\times \alpha_{S}\left(t\right)\times T_{S}\left(t\right)=\frac{\rho \mu }{d_{P}+\mu +\frac{k\left(t\right)P_{I}\left(t\right)}{N_{f}}+m}\times \beta_{S}\left(t\right)\delta w\left(t\right)P_{S}\left(t\right)\times \frac{1}{\delta w\left(t\right)+\left(1-w\left(t\right)\right)}\text{,}$$

(4) $$R_{rt}^{V}\left(t\right)=P_{V}\times \alpha_{V}\left(t\right)\times T_{V}\left(t\right)=\frac{\theta }{d_{P}+\mu +\frac{k\left(t\right)P_{I}\left(t\right)}{N_{f}}+m}\times \beta_{V}\left(t\right)P_{S}\left(t\right)\times \frac{1}{c+d_{V}}\text{,}$$

In equation (2), the first term $P_{P}$ = $\frac{\sigma }{d_{P}+\mu +\frac{k{\left(t\right)P}_{I}\left(t\right)}{N_{f}}+\sigma }$ represents the number of pigs that remain alive and infectious during the latent period per unit time. The second term $\alpha_{P}\left(t\right)=\beta_{P}\left(t\right)P_{S}\left(t\right)$, indicates the infectivity of pig-to-pig transmission, representing the expected number of susceptible pigs infected by diseased pigs per unit of time. The third term $T_{P}\left(t\right)=\frac{1}{d_{P}+\mu +\frac{k{\left(t\right)P}_{I}\left(t\right)}{N_{f}}+m}$ denotes the average infectious period of an infected pig. Similar to equation (2), in equation (3), the first term $P_{S}=\frac{\rho \mu }{d_{P}+\mu +\frac{k\left(t\right)P_{I}\left(t\right)}{N_{f}}+m}$ represents the amount of swill that becomes infectious per unit time through contamination by infected pigs. The second term $\alpha_{S}\left(t\right)=\beta_{s}\left(t\right)\delta w\left(t\right)P_{S}\left(t\right)$ represents the infectivity of swill-to-pig transmission, representing the expected number of susceptible pigs infected by contaminated swill per unit time. The third term $T_{S}\left(t\right)=\frac{1}{\delta w\left(t\right)+\left(1-w\left(t\right)\right)}$ denotes the average circulation time of contaminated swill. In equation (4), the first term $P_{V}=\frac{\theta }{d_{P}+\mu +\frac{k\left(t\right)P_{I}\left(t\right)}{N_{f}}+m}$ represents the quantity of ASFV shed per unit time by an infected pig. The second term $\alpha_{V}\left(t\right)=\beta_{V}\left(t\right)P_{S}\left(t\right)$ quantifies the infectivity of environment-to-pig transmission, indicating the expected number of susceptible pigs infected by environmental ASFV per unit time. The third term $T_{V}\left(t\right)=\frac{1}{c+d_{V}}$ represents the average survival time of ASFV in the environment.

Based on these definitions, the average number of susceptible pigs infected per unit time by a single infected pig changes dynamically over the course of the outbreak as control measures take effect. This quantity, denoted as $R_{rt}\left(t\right)$, is expressed as:

(5) $$R_{rt}\left(t\right)=R_{rt}^{P}\left(t\right)+R_{rt}^{S}\left(t\right)+R_{rt}^{V}\left(t\right)\text{.}$$

### 2.4 Parameter estimation method

Previous studies have shown that neural networks with arbitrary nonlinear activation functions can serve as universal function approximators (Pinkus, 1999). Motivated by this property, we employ a fully connected feed-forward neural network (FNN) to approximate the time-varying parameters Θ $\left(t\right)=\left[\beta_{P}\left(t\right),\beta_{S}\left(t\right),\beta_{V}\left(t\right),w\left(t\right),k\left(t\right)\right]$ in the ASF transmission model (1) that cannot be explicitly specified, thereby completing the dynamic framework.

First, we formulate a deterministic system of ordinary differential equations describing the ASF transmission process:

(6) $$\frac{dX}{dt}=f\left(X,\Theta \left(t\right)\right)\text{,}$$

where X(t) denotes the vector of state variables and Θ $\left(t\right)=\left[\beta_{P}\left(t\right),\beta_{S}\left(t\right),\beta_{V}\left(t\right),w\left(t\right),k\left(t\right)\right]$ represents the set of time-varying parameters to be estimated.

We model this relationship with a fully connected feed-forward neural network (FNN) that takes time *t* as its sole input and, after *m* hidden layers, produces the parameter vector. Let $W_{i}$ and $b_{i}$ denote the weight matrix and bias vector of the *i*-th layer, respectively, and let ϕ represent the activation function. The output of the neural network Θ(t) is then expressed as:

(7) $$\Theta \left(t\right)=\phi \left(W_{m}\phi \left(\cdots \phi \left(W_{2}\phi \left(W_{1}t+b_{1}\right)+b_{2}\right)\cdots \right)+b_{m}\right)\text{.}$$

The parameters Θ (t) output by the neural network is input into model (1), and the Runge–Kutta method is employed to numerically integrate the system, obtaining the trajectory of state variables X(t) at each time point. To simulate the dynamic transmission process of ASF in China, monthly reported new deaths and culling numbers during the epidemic were used to validate the model's feasibility. The model outputs for monthly reported new deaths and culling numbers are defined as $\hat{D}\left(t\right)$ and $\hat{C}\left(t\right)$, respectively, with corresponding calculation formulas:

(8) $$\hat{D}\left(t\right)=mP_{I}\text{,}$$

(9) $$\hat{C}\left(t\right)=k\left(t\right)P_{I}\frac{P_{S}}{N_{f}}+k\left(t\right)P_{I}\frac{P_{E}}{N_{f}}+k\left(t\right)P_{I}\frac{P_{I}}{N_{f}}\text{.}$$

To simultaneously fit the monthly reported new deaths D(t) and new culls C(t), we define the composite loss function as:

(10) $$L={MAE}_{D}+\lambda {MAE}_{C}\text{.}$$

Because the reported monthly deaths ${MAE}_{D}$ are two orders of magnitude smaller than the reported culls ${MAE}_{C}$, the squared-error term for deaths would be virtually ignored during training. We therefore introduce a scaling factor λ=0.02 to bring ${MAE}_{D}$ and ${MAE}_{C}$ to the same order of magnitude. The *MAE* is then defined as:

(11) $${MAE}_{*}=\frac{1}{N}\sum_{i=1}^{N}\left|{*}_{i}-{\hat{*}}_{i}\right|\text{,}$$

where *N* is the number of samples, ${*}_{i}$ denotes the observed value, and ${\hat{*}}_{i}$ denotes the predicted value.

(12) $${MAE}_{D}=\frac{1}{N}\sum_{i=1}^{N}\left|D_{i}-{\hat{D}}_{i}\right|\text{,}$$

(13) $${MAE}_{C}=\frac{1}{N}\sum_{i=1}^{N}\left|C_{i}-{\hat{C}}_{i}\right|\text{.}$$

**Fig. 2:**
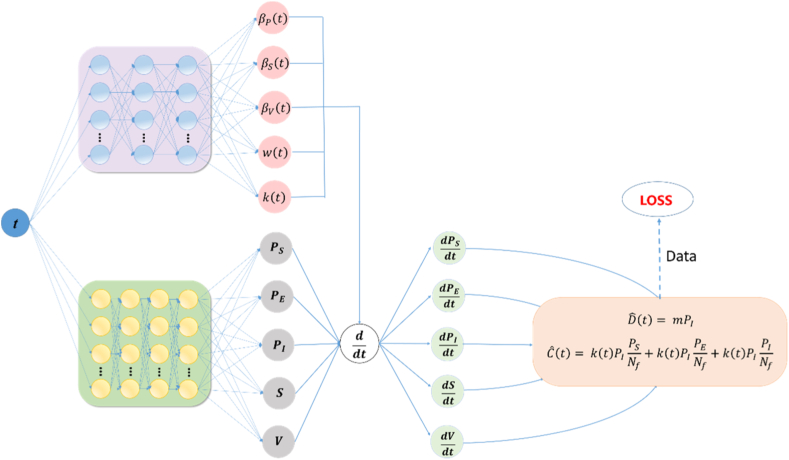
Neural-network architecture based on Model (1). Separate networks represent the state variables (green-shaded region) and the time-varying parameters (purple-shaded region) of Model (1). $\frac{d}{dt}$ denotes the automatic-differentiation operator.

Finally, we trained the neural network to minimize the loss function (10), simultaneously learning network parameters and inferring unknown time-varying parameters of model (1) (Fig. 2). Model fitting and parameter estimation were performed using the FNN algorithm, as illustrated schematically in Fig. 2. The implementation was developed using PyTorch, an open-source library for deep-learning computations (Subramanian, 2018). The neural network employs ReLU activation functions, and the composite loss function (10) is optimized using the Adam optimizer (Kingma & Ba, 2017). To enhance performance, we utilized the Optuna hyperparameter optimization framework (Akiba et al., 2019) to determine the optimal number of hidden layers, hidden units per layer, and initial learning rate. The optimal architecture comprised three hidden layers, each containing 64 nodes, with an initial learning rate of 0.001. The network was retrained using these optimal parameters, which were then saved for subsequent numerical simulations.

## 3 Results

### 3.1 Estimation of time-varying parameters

**Fig. 3:**
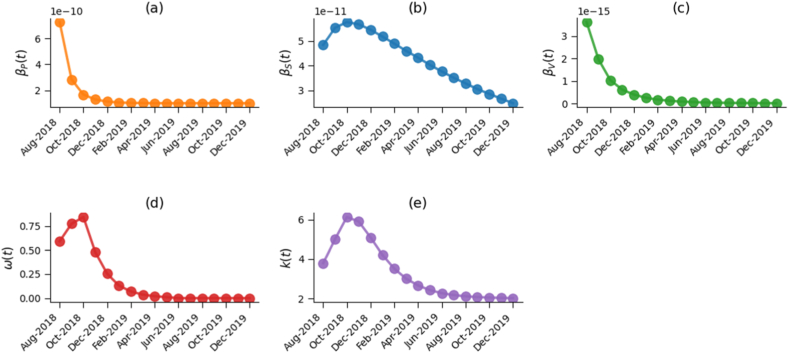
Estimated temporal trajectories of key time-varying parameters: pig-to-pig transmission rate ($\beta_{P}\left(t\right)$), swill-to-pig transmission rate ($\beta_{S}\left(t\right)$), environment-to-pig transmission rate ($\beta_{V}\left(t\right)$), proportion of swill used for pig feeding (w(t)), and average number of farms subjected to sanitary culling after detection (k(t)).

Using monthly ASF mortality and culling data from China (August 2018-December 2019), we estimated the temporal trajectories of five time-varying parameters: the effective transmission rates from infectious pigs ($\beta_{P}\left(t\right)$), contaminated swill ($\beta_{S}\left(t\right)$), and environmental virus ($\beta_{V}\left(t\right)$) to susceptible pigs, the proportion of total swill diverted to pig feeding (w(t)), and the average number of farms subjected to sanitary culling after detection (k(t)). As shown in Fig. 3, the three transmission pathways exhibit distinct temporal patterns with clear epidemiological interpretations. Specifically, $\beta_{S}\left(t\right)$ increased rapidly in the early stage of the epidemic, rising from approximately 4.85e-11 to an initial peak of 5.77e-11 in October 2018, before gradually declining to 2.49e-11 by the end of the study period. This trend reflects the widespread use of untreated swill prior to the nationwide swill-feeding ban and the subsequent strengthening of feed management policies. In contrast, both $\beta_{P}\left(t\right)$ and $\beta_{V}\left(t\right)$ started at relatively high levels ($\beta_{P}\left(t\right)$ ≈7.27e-10, $\beta_{V}\left(t\right)$ ≈3.63e-15 in August 2018, respectively), decreased sharply within the first few months following the outbreak, falling to 1.00e-10 and 7.0719e-17 by May 2019, and then remaining at low, stable levels, which is consistent with the rapid implementation of emergency interventions, including movement restrictions, enhanced farm biosecurity, and intensified environmental disinfection. Meanwhile, the control-related parameters w(t) and k(t) increased significantly during the epidemic peak, reaching 0.85 and 6.13 in October 2018, and then gradually declined, reflecting the escalation and subsequent relaxation of control intensity as ASF transmission was progressively brought under control.

**Fig. 4:**
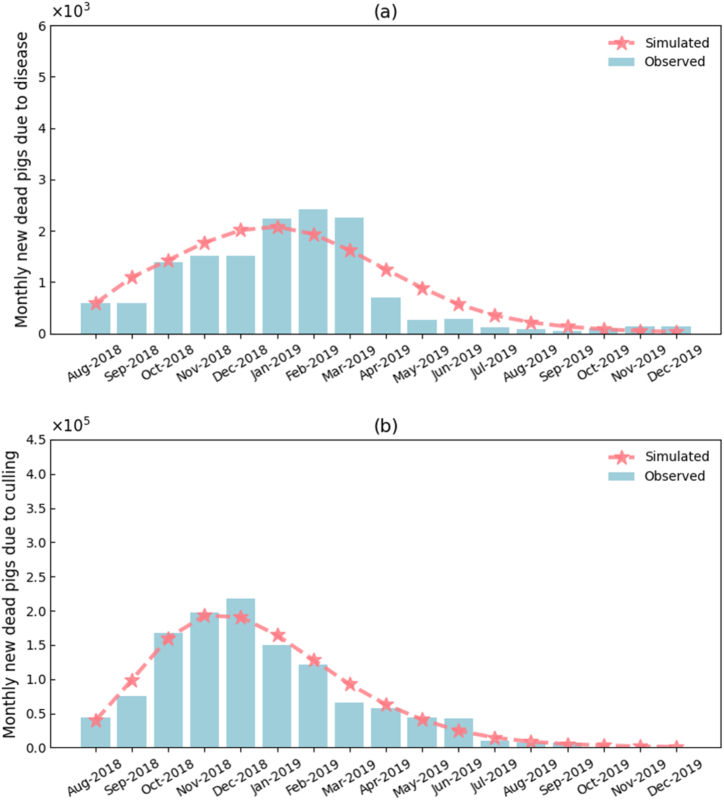
Model fit to monthly numbers of new ASF deaths and new culls in China from August 2018 to December 2019.

Fig. 4 compares the model-fitted monthly numbers of new ASF deaths and new culls with the corresponding observed values. Quantitative metrics indicated a mean absolute error (MAE) of 276.32 and a coefficient of determination ($R^{2}$) of 0.82 for new deaths, and an MAE of 9099.50 with an $R^{2}$ = 0.97 for new culls. These accuracy indicators demonstrate that the model satisfactorily reproduces both the epidemic surge and the dynamic implementation of control measures in China, thereby providing a robust parameterized basis for subsequent scenario projections and strategy evaluations.

### 3.2 Real-time reproduction number

**Fig. 5:**
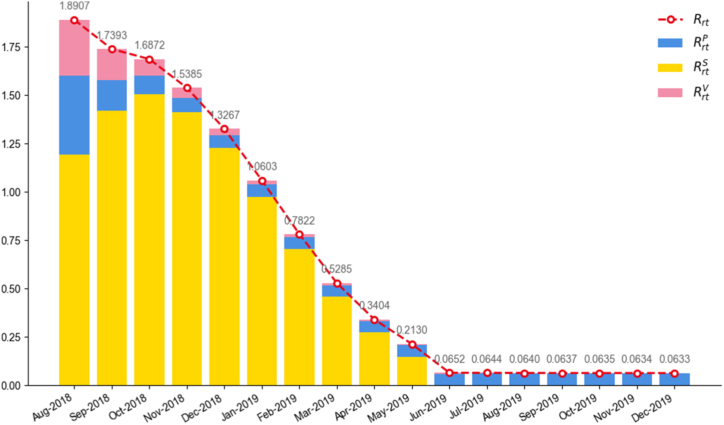
Stacked bar chart of the real-time reproduction number $R_{rt}\left(t\right)$ of ASF in China from August 2018 to December 2019. The blue segment represents the average number of susceptible pigs infected by one infectious pig during its infectious period $\left(R_{rt}^{P}\left(t\right)\right)$; the yellow segment represents the average number of susceptible pigs infected per kilogram of contaminated swill during its circulation time $\left(R_{rt}^{S}\left(t\right)\right)$; and the pink segment represents the average number of susceptible pigs infected per unit of environmental ASFV during its survival period $\left(R_{rt}^{V}\left(t\right)\right)$.

Based on the definition of the time-varying reproduction number $R_{rt}\left(t\right)$) presented in Section 2.3, Fig. 5 shows a stacked bar chart of ASF $R_{rt}\left(t\right)$ in China from August 2018 to December 2019. Overall, $R_{rt}\left(t\right)$ exhibited a gradual downward trend and stabilized in the later stages, indicating visually that the epidemic was effectively contained under the combined effects of multiple control measures. Specifically, at the onset of the epidemic (August 2018), $R_{rt}\left(t\right)$ peaked at 1.8907, which is lower than the estimate $R_{0}=2.45$ reported in our previous study (J. Li et al., 2024). Despite this difference, the peak value remained significantly above the epidemic threshold of 1, indicating high transmissibility. As layered control measures were progressively implemented and refined, $R_{rt}\left(t\right)$ steadily declined, reaching 0.7822 by February 2019, a value below 1, signifying effective curtailment of transmission and transition of the outbreak into a controllable phase. After June 2019, $R_{rt}\left(t\right)$ stabilized at approximately 0.0650, a level close to zero, signifying entry into a phase of stable, routine control with minimal transmission risk. Overall, the temporal evolution of $R_{rt}\left(t\right)$ is consistent with our previous findings (J. Li et al., 2024), although the rate of change in the present study is relatively slower.

During the early epidemic phase, all three pathways substantially contributed to transmission: pig-to-pig contact yielded $R_{rt}^{P}\left(t\right)=0.4067$, swill-borne transmission $R_{rt}^{S}\left(t\right)=1.1946$, and environmental virus $R_{rt}^{V}\left(t\right)=0.2894$. Notably, the reproduction number via the swill route was considerably higher than those of the other two, confirming that contaminated swill was the dominant transmission driver at the epidemic's outset.

Direct pig-to-pig contact and environmental ASFV also provided non-negligible secondary routes. As control measures intensified, the reproductive contribution of each pathway declined significantly. Notably, $R_{rt}^{S}\left(t\right)$ remained at zero from June 2019 onward, confirming that the swill-feeding ban completely severed this transmission chain, while $R_{rt}^{V}\left(t\right)$ stabilized near 0.002, illustrating successful environmental disinfection efforts in minimizing virus circulation. The pig-to-pig component $R_{rt}^{P}\left(t\right)$ stabilized at a low level of approximately 0.061, further indicating that continuous stamping-out and sustained biosecurity measures remain essential for interrupting animal-to-animal ASF transmission.

### 3.3 Numerical simulations

To systematically evaluate the impact of zoning prevention and control policies on ASF transmission in China, the entire country is abstracted into two regions in the model simulations in this section. This division is not based on actual geographic or administrative boundaries, but represents a theoretical and scenario-based abstraction of policy implementation units, aiming to focus on the effect of the zoning mechanism itself.

All simulation scenarios strictly follow the control variable principle: when evaluating the effect of a specific prevention and control measure (e.g., timing of zoning implementation, regional division ratio, biosecurity level, disposal intensity of diseased and dead pigs, enforcement strength of banning swill feeding, etc.), all other parameters and conditions remain unchanged. For example, when analyzing the impact of regional division, the two regions differ only in the proportional allocation of initial state variables. Beyond this, the content, implementation intensity, and implementation timing of prevention and control measures are completely identical between the two regions, and all epidemiological processes and intervention mechanisms remain the same. This design can effectively isolate and quantify the independent effect of zoning policy configuration while avoiding confounding interference from spatial heterogeneity assumptions, thereby ensuring the reliability and interpretability of the simulation results.

Using the previously developed non-autonomous dynamic model, we conducted comparative simulations under various scenarios. Using a model scenario without zonal policies as a baseline, we analyzed transmission dynamics and control effectiveness across the following three scenarios:

Scenario S1 (zonal control policy evaluation): Systematically varies initiation timing and within-zone farm-density ratios to quantify their effects on transmission.

Scenario S2 (single-intervention efficacy): Independently manipulates producer biosecurity awareness, intensity of safe carcass disposal, and enforcement strength of the swill-feeding ban to evaluate the standalone effectiveness of each measure.

Scenario S3 (combined-strategy synergy): First, jointly optimizes initiation timing and within-zone farm-density ratios under zonal policies. Second, simultaneously varies biosecurity awareness, carcass disposal intensity, and swill-ban enforcement to evaluate the integrated effectiveness of combined measures.

For clarity, all scenario-specific parameters, including initial conditions, transmission rates (β), swill-feeding ban intensity (ω), and carcass disposal intensity (k) (Appendix A.1).

#### 3.3.1 Targeted evaluation of zoned prevention and control policies

To evaluate the influence of core components in zoned prevention-and-control policies-specifically, implementation time (T) and zonal proportion, on ASF transmission, this study fixed key parameters within Zone I and Zone II, including route-specific transmission rates ($\beta_{P}$, $\beta_{S}$, $\beta_{V}$), swill-feeding-ban enforcement intensity (ω); and harmless-treatment intensity (k)). By doing so, we focused explicitly on how variations in T and zonal proportion modulate monthly incident infections and cumulative epidemic mortality. The zonal proportion was set such that the initial value of each state variable in Zone I accounts for ${SV}_{I}=\frac{2}{3}SV$, and in Zone II for ${SV}_{I}=\frac{1}{3}SV$, where SV represents the five core state variables (e.g., susceptible pigs, infected pigs). All subsequent zonal proportion configurations follow this convention.

(1) **Impact of zoned control implementation timing**

**Fig. 6:**
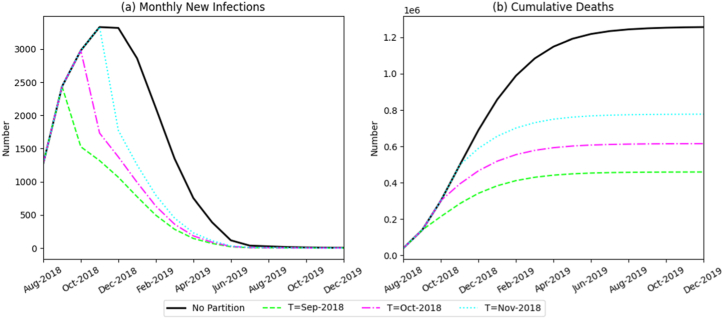
Simulation results of the monthly number of newly infected pigs and the total death toll under different zoned-implementation periods.

Fig. 6 shows that under the baseline scenario without interventions, the ASF outbreak spread rapidly, peaking at 2979 infected pigs and resulting in 1.256 million cumulative deaths, underscoring the severe consequences of an uncontrolled epidemic.

In contrast, implementing zoned control policies substantially reduced viral transmission, with the extent of reduction closely linked to the timing of implementation. Earlier intervention produced the strongest effect. When zoning began in September 2018, peak infections decreased to 2435 pigs, and cumulative mortality declined to 458,800 head, representing more than a 55% reduction compared with the baseline. Delaying implementation to October or November 2018 increased cumulative deaths to 614,900 and 776,900 head, respectively, demonstrating a clear decline in control effectiveness. These results indicate that the initiation date of zoned prevention is a critical determinant of epidemic outcomes. Early implementation minimizes losses across the swine industry.

(2) Impact of zonal proportion

**Fig. 7:**
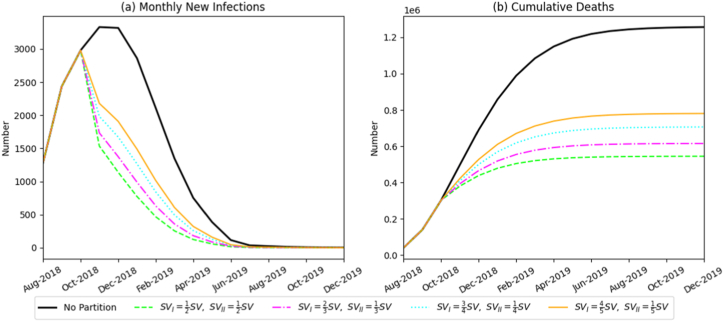
Simulation results of the monthly number of newly infected pigs and the total death scale under different zoned-proportion scenarios.

With the start of zoned control fixed at October 2018, we assessed how the allocation proportion between Zone I and Zone II influences outbreak dynamics (Fig. 7). As the initial pig population distribution between the two zones became more balanced, the monthly incidence peak occurred earlier, the speed of viral spread slowed, and cumulative mortality decreased steadily from 780,400 to 544,500 head. These results demonstrate that a more balanced zonal distribution improves overall containment effectiveness.

#### 3.3.2 Evaluation of single-strategy effectiveness

To explore the independent regulatory effects of single measures, such as protection awareness, harmless-treatment intensity, and the implementation intensity of the swill-feeding prohibition, on ASF transmission, we fixed the parameters related to zoned prevention and control (implementation time T = October 2018, and the zoned proportion is ${SV}_{I}=\frac{2}{3}SV,{SV}_{II}=\frac{1}{3}SV$). We then analyzed differences in how these individual measures influence the monthly number of newly infected pigs and the overall epidemic death scale. The results are as follows:

(1) Effect of increased public awareness on ASF control

To represent improvements in public awareness, we assume that enhanced protective awareness exerts a consistent influence across multiple transmission pathways. Accordingly, the transmission coefficients $\beta_{P}$, $\beta_{S}$, and $\beta_{V}$ are assumed to vary proportionally. A unified scaling parameter $\beta =\left(\beta_{P},\beta_{S},\beta_{V}\right)$ is introduced to characterize their joint modulation under different awareness levels.

**Fig. 8:**
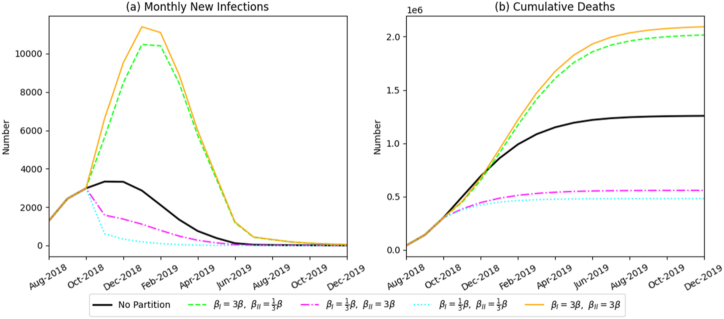
Simulation results of monthly new infections and cumulative deaths under varying levels of biosecurity awareness.

By simultaneously adjusting transmission rate parameters for $\beta_{P}$, $\beta_{S}$, $\beta_{V}$, we simulated graded improvements in on-farm biosecurity awareness. Fig. 8 illustrates that enhanced awareness significantly improves ASF containment. Under conditions of low awareness, the epidemic expanded rapidly, peaking at 11,999 infected pigs in January 2019 and causing 2.09 million cumulative deaths, surpassing the no-intervention baseline. In contrast, a high-awareness scenario reduced cumulative mortality to 480,386 pigs, an outcome superior to most single-intervention strategies. These results highlight the crucial role of behavioral changes at the producer level in emergency disease management.

(2) Efficacy of harmless-treatment intensity

**Fig. 9:**
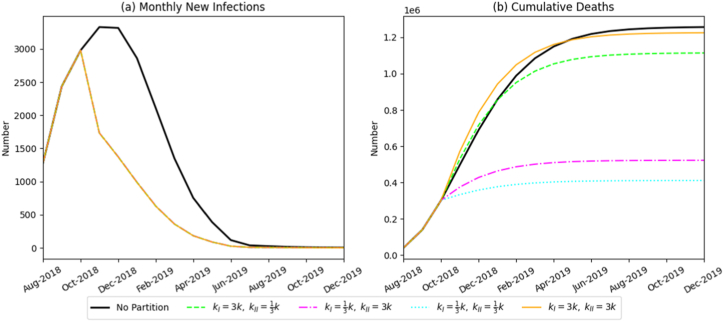
Simulation results of monthly new infections and cumulative deaths under varying intensities of harmless treatment.

Unlike other interventions, harmless-treatment intensity significantly influenced only total epidemic mortality, with minimal impact on monthly incident infections (Fig. 9). Specifically, doubling treatment intensity increased cumulative deaths to 1.225 million pigs, whereas insufficient treatment intensity reduced mortality to 410,600 pigs. This indicates a positive correlation between treatment intensity and overall death toll.

(3) Efficacy of the swill-feeding ban

**Fig. 10:**
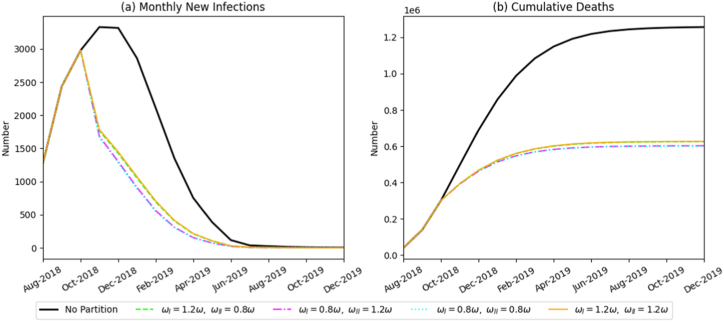
Simulation results of monthly new infections and cumulative deaths under varying enforcement intensities of the swill-feeding ban.

Compared to a no-zoning scenario, implementing the swill-feeding ban within a zonal-control framework provided notable benefits. However, once zoning was established, further increases in enforcement intensity yielded only marginal improvements (Fig. 10). Under zonal conditions, cumulative mortality varied slightly between 600,600 and 626,600 pigs across different enforcement levels-a difference of less than 4%, lacking statistical significance.

#### 3.3.3 Synergistic evaluation of combined prevention and control strategies

Under scenarios combining zonal control with protective awareness, harmless-treatment intensity, and swill-feeding prohibition, integrated prevention efficacy was markedly superior to that of any single measure. The detailed analysis is presented below.

**Fig. 11:**
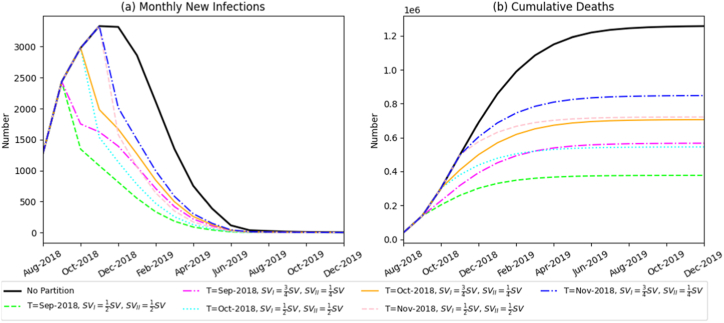
Simulation results showing the monthly number of newly infected pigs and total mortality under various combinations of zoned prevention and control measures.

In this section, we conducted scenario simulations combining various start dates and zonal proportions of the partitioned prevention-and-control policy. Transmission rates for each route ($\beta_{P}$, $\beta_{S}$, $\beta_{V}$), enforcement intensity of the swill feeding ban (ω), and intensity of harmless treatment (k) were held constant in Zone I and Zone II to examine the synergistic optimization of the policy's core elements; the results are shown in Fig. 11. When a balanced (1:1) partition was introduced in September 2018, the peak number of newly infected pigs decreased to 2,435, and total mortality was limited to 376,800 pigs. This balanced ratio resulted in a greater reduction in infected cases and significantly lower mortality compared to a 3:1 ratio. Early implementation of zonal control consistently restrained ASF spread. Specifically, earlier implementation combined with a more balanced partition ratio yielded superior control outcomes, reducing both the peak monthly incidence and overall mortality, thus markedly improving the efficiency and effectiveness of ASF containment.

**Fig. 12:**
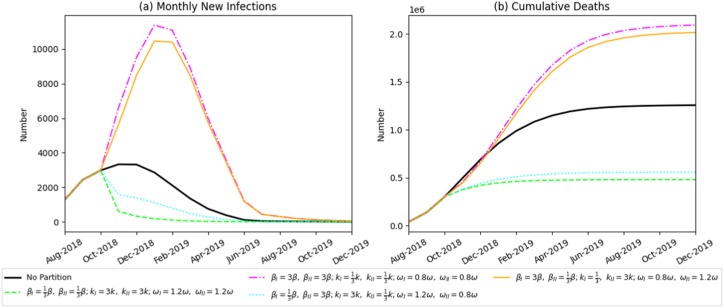
Simulation results showing the monthly number of newly infected pigs and total mortality under various combinations of other policies.

Next, we fixed zonal control parameters with the policy starting in October 2018 and the partition ratio set as ${SV}_{I}=\frac{2}{3}SV,{SV}_{II}=\frac{1}{3}SV$, then examined the combined effect of multiple measures. Results are shown in Fig. 12. When Zone I and Zone II simultaneously intensified the three interventions, raising biosecurity awareness, optimizing harmless treatment, and strictly enforcing the swill-feeding ban, total mortality decreased to 480,386 pigs, and incident infections declined significantly. This outcome far surpassed that achieved by any single measure alone. Conversely, when all three measures were inadequately implemented, the epidemic approached an uncontrolled state, and total mortality reached 2,092,800 pigs. These findings demonstrate that the synergistic application of multiple control measures provides a distinct advantage by blocking transmission along multiple dimensions and throughout the entire transmission chain, thereby generating a robust combined inhibitory effect on ASF spread.

### 3.4 Uncertainty and sensitivity analysis

**Fig. 13:**
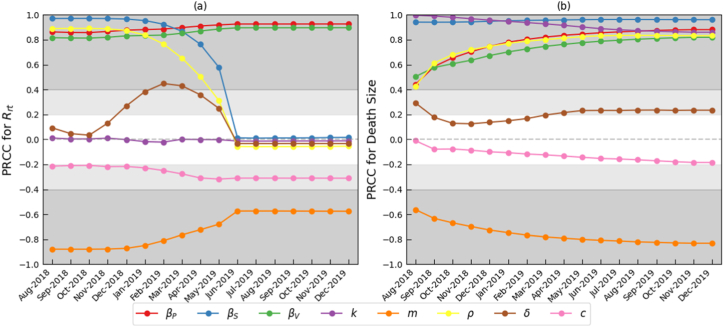
Sensitivity analysis results for the real-time reproduction number $R_{rt}\left(t\right)$ and total mortality over time.

In this section, Latin hypercube sampling (LHS) was employed to generate 1000 parameter sets, and partial rank correlation coefficients (PRCC) were calculated between each input variable and the two output variables, real-time reproduction number $R_{rt}\left(t\right)$ and total mortality, to quantify parameter influence (Marino et al., 2008). Due to the lack of prior distribution information and validated data for input parameters, all variables were assumed to follow uniform distributions. Constant parameters were allowed to vary within 80% to 120% of their estimated values as shown in Table 2, whereas time-dependent parameters were assigned ranges from their observed minimum to maximum values. Sensitivity analysis results are presented in Fig. 13.

Fig. 13(a) illustrates PRCC analysis of key model parameters concerning the temporal dynamics of the real-time reproduction number $R_{rt}\left(t\right)$. Throughout the study period, the direct pig-to-pig transmission rate $\beta_{P}\left(t\right)$ and the environmental ASFV transmission rate $\beta_{V}\left(t\right)$ consistently exhibited strong positive correlations with $R_{rt}\left(t\right)$ (|PRCC|≥0.8), indicating their dominant influence on disease transmission potential. In contrast, the swill-mediated transmission rate $\beta_{S}\left(t\right)$ showed clear time-dependent sensitivity. From August to December 2018, its PRCC remained above 0.8, reflecting a strong positive association and highlighting its major role during the early outbreak phase. However, from January 2019 onward, the PRCC gradually declined to approximately zero, indicating a significant weakening in the relative importance of swill-borne transmission, consistent with restrictive swill-feeding policies. The case fatality rate of infected pigs *m* consistently maintained a strong negative correlation with $R_{rt}\left(t\right)$ (|PRCC|≥0.4). Notably, the conversion rate of infected pigs to contaminated water, ρ, shifted from a strong positive to a weak negative correlation over time, while the consumption rate of swill by pigs, δ, transitioned from weak positive to moderate positive before declining to weak negative correlation, patterns aligning with the enforcement of swill-feeding bans. In addition, the ASFV clearance rate *c* and the average number of culled farms *k* showed only weak correlations with $R_{rt}\left(t\right)$ (|PRCC|≤0.4) throughout the study period.

Fig. 13(b) presents PRCC results for key model parameters concerning the temporal evolution of total mortality, revealing sensitivity patterns that differ somewhat from those observed for $R_{rt}\left(t\right)$. Parameters $\beta_{P}\left(t\right)$, $\beta_{S}\left(t\right)$, $\beta_{V}\left(t\right)$, k, m*,* and ρ consistently demonstrated strong correlations (|PRCC|≥0.4). Among them, $\beta_{P}\left(t\right)$, $\beta_{S}\left(t\right)$, $\beta_{V}\left(t\right)$, and ρ remained positively correlated, with correlation strengths increasing over time. Parameter *k* also consistently maintained a strong positive correlation, although its strength declined gradually over the years. In contrast, *m* exhibited a consistent negative correlation, with the correlation magnitude increasing over time. Additionally, parameters *c* and δ showed weak correlations throughout the study period.

## 4 Discussion

Existing studies modeling ASF transmission dynamics predominantly rely on classical SIR or SEIR frameworks and their variants, such as SEICD and SEID. Parameter estimation in these models typically employs static, fixed values (Ali et al., 2024; Chen & Hsieh, 2012; Fatmawati et al., 2020; Gong et al., 2023; Jhutty & Hernandez-Vargas, 2022; Kamrujjaman & Mohammad, 2024; Kang & Li, 2024; Naaly et al., 2024; Rahman et al., 2019; Srivastav & Ghosh, 2021). Although these models effectively illustrate macroscopic epidemic trajectories under various control scenarios, they fail to accurately capture the dynamic features of transmission, particularly gradient changes in intervention intensity and real-time adjustments in producer behavior. In practice, key parameters such as infection rates, culling proportions, and swill usage ratios fluctuate significantly due to policy adjustments and seasonal shifts in market supply and demand. Such dynamic influences cannot be adequately reflected by conventional static parameter assumptions. Furthermore, many studies assess ASF transmission potential by calculating the basic reproduction number $R_{0}$ (Ayihou et al., 2024; Hsu et al., 2025; Mai et al., 2022; Shi & Zhang, 2024), which characterizes transmissibility under the assumption of a fully susceptible population and is typically interpreted as an early-phase, time-invariant summary metric. As such, $R_{0}$ is not designed to capture temporal changes in transmission under evolving interventions, and it often does not explicitly disentangle the contributions of distinct transmission routes (e.g., direct contact, swill feeding, and environmental contamination). Consequently, relying solely on $R_{0}$ may provide limited resolution for quantitatively evaluating the targeted effectiveness of specific control measures.

To address these research gaps, we developed a coupled model combining deep neural networks (FNN) and non-autonomous transmission dynamics. The neural network component dynamically estimates critical model parameters, while the structural design incorporates interactions among pigs, swill, and the environment, significantly enhancing the model's capacity to capture time-varying transmission dynamics. Based on dynamic parameter estimation, transmission efficiency across all three routes was highest during the initial epidemic wave (August 2018). Following the gradual implementation of various prevention and control measures, transmission intensity showed a stepwise decline. Notably, parameters linked to control intensity and culling proportion rose sharply during peak epidemic periods (October-November 2018) and then gradually declined as the epidemic subsided. This accurately reflects real-world conditions, transitioning from intensive control during critical periods to precise regulation under normalized management. To overcome limitations associated with conventional evaluation indicators, our study proposed route-specific, real-time reproduction numbers under zonal prevention and control conditions, providing improved interpretability in quantifying control effectiveness. Findings indicated that during the initial outbreak (August 2018), ASF posed a high transmission risk through all three transmission channels. Among these, swill-mediated transmission predominated, followed by direct pig-to-pig contact and environmental transmission, consistent with previous empirical research (Gao et al., 2021). After implementing combined interventions, including the prohibition of swill feeding, intensified environmental disinfection, and enhanced biosecurity, the real-time reproduction numbers for all three transmission pathways markedly decreased. The epidemic became controllable by February 2019, stabilizing after June 2019. Through this dynamic approach, we verified the overall efficacy of control measures and quantified the differential impacts of individual interventions on reducing ASF transmission.

Quantitative analysis based on multi-scenario simulations demonstrates that the timing, intensity, and combination of control measures have a decisive impact on the epidemic trajectory. These findings provide essential theoretical support and practical guidance for establishing a scientifically sound and efficient prevention and control system. To begin with, the success of zoned prevention and control relies on timely and well-designed implementation. Being the central spatial intervention, the initiation timing of zoning fundamentally affects control outcomes. Our results indicate that zoned measures implemented at the beginning of the epidemic significantly influence overall outcomes; once this critical phase is missed, subsequent interventions yield considerably diminished results, as the virus is already spreading. This trend highlights the fundamental principle of early detection and early response in epidemic management and aligns theoretically with the so-called window-period effect in infectious disease control (Daniel Beltran-Alcrudo et al., 2017). In addition to timing, the rationality of zoning strategies also critically affects effectiveness. Our simulations revealed that the efficiency of blocking cross-regional transmission is directly associated with balanced zonal proportions. Highly clustered infections within zoning plans tend to result in increased local transmission and higher inter-regional importation risks. Conversely, a moderate zoning strategy ensures spatial concentration of infected populations, achieving both local containment and regional isolation. These findings confirm that successful zoning implementation requires balancing timeliness and scientific design, and this combination is essential for maximizing spatial intervention effectiveness. Second, individual control measures vary significantly in effectiveness, with distinct mechanisms and implementation contexts. Behavioral interventions, prompted by increased biosecurity awareness, including intensified on-farm biosafety measures, are the cornerstone of the national control system. These measures are indispensable for controlling epidemic spread via multiple routes, as they minimize exposure risks to susceptible populations through proactive behavioral changes. A critical intervention focus is the intensity of harmless disposal, which directly impacts infection sources. The effectiveness of this intervention exhibits a clear cost-benefit trade-off. While disposal intensity can rapidly reduce infectious reservoirs, excessive increases significantly escalate control costs without corresponding epidemic reduction. Therefore, a dynamic adjustment mechanism for harmless disposal intensity is required, considering crucial indicators such as the rate of epidemic spread and infection levels to balance control effectiveness and cost efficiency. Conversely, the independent protective effect of prohibiting swill feeding is relatively modest. Although this measure effectively eliminates one transmission channel when combined with zoned control, minor variations in enforcement intensity yield only marginal benefits and cannot independently satisfy core containment requirements. This outcome primarily arises from temporal misalignment, as significant swill-borne risks had already been reduced by intensive swill-feeding prohibition before zoning. Subsequent intensity adjustments post-zoning has minimal influence on overall epidemic progression. This finding suggests that the effectiveness of individual measures must be assessed together with timing and situational context and not implemented separately outside the integrated system. Additionally, multi-scenario simulations demonstrate that the combined strategy of "early implementation and balanced zoning" consistently achieves optimal control performance. This approach results in optimal spatial regulation and source control, reducing both epidemic peaks and overall losses, thereby reaffirming that timely implementation and scientifically valid structures are essential for successful spatial interventions. Importantly, the study confirms that the concurrent application of multiple control measures produces strong synergistic effects, and the absence of essential interventions may drastically reduce overall control efficiency. Strengthening biosecurity awareness exemplifies this, as insufficient awareness can render the entire control framework ineffective despite other interventions, leading to losses potentially exceeding those under no intervention. This phenomenon reflects the short-board effect in control systems, whereby overall effectiveness depends on the weakest intervention component. Although this study provides scientific guidance for the prevention and control of ASF in China, several limitations remain. First, parameter estimation relied substantially on official reports and statistical data, meaning that data completeness and accuracy directly influenced the results. Specifically, underreporting or delayed reporting during early epidemic stages could bias models describing initial transmission dynamics. Second, complex real-world constraints, such as regional differences in policy enforcement, variability in farmers’ awareness and compliance capabilities, and resource availability, were not considered (J. Li et al., 2023). These factors may significantly impact the practical effectiveness of proposed control measures. Future research should integrate broader data sources and account for detailed real-world constraints to enhance the practical applicability of findings.

## 5 Conclusion

This paper examines the practical aspects of ASF transmission and control within China by employing neural networks and dynamic modeling to develop a quantifiable analytical model. It evaluates the effectiveness of zoned prevention and control measures, while a combination of sensitivity analysis and dynamic simulation identifies key factors influencing ASF transmission in China. The results offer quantitative evidence and theoretical recommendations for the scientific optimization of management strategies. Findings underline that the efficacy of control measures is highly time-sensitive and synergistic, factors that critically determine the epidemic's trajectory.

Initiating zoned control at an early stage and significantly improving producers’ biosecurity awareness constitute the primary strategic solutions for reducing epidemic losses through spatial isolation and source-risk reduction. Maintaining a moderate level of harmless disposal intensity and enforcing a ban on swill-feeding serve as supportive measures. Their intensity should be calibrated based on real-time epidemic dynamics or combined with core measures. Additionally, simultaneous and strict implementation of multiple interventions is fundamental for successful ASF control. Implementing individual interventions separately or omitting critical measures markedly reduces overall system effectiveness. Sensitivity analyses further confirm the dominant role of core parameters in influencing epidemic scale and peak infection numbers, supporting the empirical prioritization of control strategies. In summary, ASF control requires a three-dimensional approach incorporating accurate timing, scientific design, and integrated implementation. By capturing the early intervention window, optimizing key operational parameters such as zoning, and strengthening synergistic interactions among various measures, it is possible to effectively prevent transmission across multiple levels and stages, thereby significantly enhancing overall control effectiveness. The quantitative evaluation approach and integrated control system developed in this study provide technical guidance for improving the scientific accuracy and effectiveness of animal disease prevention and control in China. They also offer a transferable technical strategy and practical framework for responding to international threats from significant transboundary animal diseases such as ASF. The model requires further adjustments to address diverse situations in future applications, and the establishment of differentiated control strategies tailored to various farming systems and regional geographic characteristics, thus enabling more targeted prevention and control practices.

## CRediT authorship contribution statement

**Juan Li:** Writing – review & editing, Writing – original draft, Methodology, Funding acquisition, Formal analysis, Conceptualization. **Junhui Zhang:** Writing – original draft, Visualization, Validation, Software, Methodology. **Lu Gao:** Writing – review & editing, Validation, Data curation. **Shubo Li:** Writing – review & editing, Methodology, Conceptualization. **Huaiping Zhu:** Writing – review & editing, Supervision, Conceptualization.

## Declaration of competing interest

The authors declare that we have no known competing financial interests or personal relationships that could have appeared to influence the work reported in this paper.

## Figures and Tables

**Table:** 

Scenario	Description	Fixed Parameters	Simulation Parameters	Epidemic Peak	Total Death Toll
Baseline Reference	Model without zoned prevention and control	See Table 2	See Table 2	3330	1256592
Scenarios S1: Zoning Policy-related Parameter Changes	Policy Implementation Timeline	$\beta_{I}=\beta ,\beta_{II}=\beta \text{;}$ $w_{I}=w,w_{II}=w\text{;}$ $k_{I}=k,k_{II}=k\text{;}$ ${SV}_{I}=\frac{2}{3}SV,{SV}_{II}=\frac{1}{3}SV$	T = Sep-2018	2435	458824
			T = Oct-2018	2979	614921
			T = Nov-2018	3330	776926
	Parameter variation for Zoning Proportion	T = Oct-2018 $\beta_{I}=\beta ,\beta_{II}=\beta \text{;}$ $w_{I}=w,w_{II}=w\text{;}$ $k_{I}=k,k_{II}=k$	${SV}_{I}=\frac{1}{2}SV,{SV}_{II}=\frac{1}{2}SV$	2979	544501
			${SV}_{I}=\frac{2}{3}SV,{SV}_{II}=\frac{1}{3}SV$	2979	614921
			${SV}_{I}=\frac{3}{4}SV,{SV}_{II}=\frac{1}{4}SV$	2979	705939
			${SV}_{I}=\frac{4}{5}SV,{SV}_{II}=\frac{1}{5}SV$	2979	780410
Scenario S2: Evaluation of the Effectiveness of a Single Prevention and Control Strategy	Parameter variation for awareness-strengthening intensity	T = Oct-2018 $w_{I}=w,w_{II}=w\text{;}$ $k_{I}=k,k_{II}=k\text{;}$ ${SV}_{I}=\frac{2}{3}SV,{SV}_{II}=\frac{1}{3}SV$	$\beta_{I}=3\beta ,\beta_{II}=\frac{1}{3}\beta$	10476	2015584
			$\beta_{I}=\frac{1}{3}\beta ,\beta_{II}=3\beta$	2979	557626
			$\beta_{I}=\frac{1}{3}\beta ,\beta_{II}=\frac{1}{3}\beta$	2979	480386
			$\beta_{I}=3\beta ,\beta_{II}=3\beta$	11399	2092824
	Parameter variation for harmless-treatment execution intensity	T = Oct-2018 $\beta_{I}=\beta ,\beta_{II}=\beta \text{;}$ $w_{I}=w,w_{II}=w\text{;}$ ${SV}_{I}=\frac{2}{3}SV,{SV}_{II}=\frac{1}{3}SV$	$k_{I}=3k,k_{II}=\frac{1}{3}k\text{;}$	2979	1113545
			$k_{I}=\frac{1}{3}k,k_{II}=3k$	2979	522315
			$k_{I}=\frac{1}{3}k,k_{II}=\frac{1}{3}k$	2979	410663
			$k_{I}=3k,k_{II}=3k$	2979	1225197
	Parameter variation for swill-feeding prohibition policy implementation intensity	T = Oct-2018 $\beta_{I}=\beta ,\beta_{II}=\beta \text{;}$ $k_{I}=k,k_{II}=k\text{;}$ ${SV}_{I}=\frac{2}{3}SV,{SV}_{II}=\frac{1}{3}SV$	$w_{I}=1.2w,w_{II}=0.8w$	2979	624883
			$w_{I}=0.8w,w_{II}=1.2w$	2979	602293
			$w_{I}=0.8w,w_{II}=0.8w$	2979	600557
			$w_{I}=1.2w,w_{II}=1.2w$	2979	626619
**Scenario S3: Synergistic Evaluation of Combined Prevention and Control Strategies**	Parameter variation for timing and zonal proportion of regional prevention and control policies	T = Sep-2018 $\beta_{I}=\beta ,\beta_{II}=\beta \text{;}$ $w_{I}=w,w_{II}=w\text{;}$ $k_{I}=k,k_{II}=k$	${SV}_{I}=\frac{1}{2}SV,{SV}_{II}=\frac{1}{2}SV$	2435	376833
			${SV}_{I}=\frac{3}{4}SV,{SV}_{II}=\frac{1}{4}SV$	2435	566641
		T = Oct-2018 $\beta_{I}=\beta ,\beta_{II}=\beta \text{;}$ $w_{I}=w,w_{II}=w\text{;}$ $k_{I}=k,k_{II}=k$	${SV}_{I}=\frac{1}{2}SV,{SV}_{II}=\frac{1}{2}SV$	2979	544501
			${SV}_{I}=\frac{3}{4}SV,{SV}_{II}=\frac{1}{4}SV$	2979	705939
		T = Nov-2018 $\beta_{I}=\beta ,\beta_{II}=\beta \text{;}$ $w_{I}=w,w_{II}=w\text{;}$ $k_{I}=k,k_{II}=k$	${SV}_{I}=\frac{1}{2}SV,{SV}_{II}=\frac{1}{2}SV$	3330	721645
			${SV}_{I}=\frac{3}{4}SV,{SV}_{II}=\frac{1}{4}SV$	3330	847473
	Parameter variations in the combined intensity of awareness enhancement, harmless treatment execution, and swill-feeding prohibition policy implementation	T = Oct-2018 ${SV}_{I}=\frac{2}{3}SV,{SV}_{II}=\frac{1}{3}SV$	$\beta_{I}=\frac{1}{3}\beta ,\beta_{II}=\frac{1}{3}\beta \text{;}$ $k_{I}=3k,k_{II}=3k\text{;}$ $w_{I}=1.2w,w_{II}=1.2w$	2979	480386
			$\beta_{I}=3\beta ,\beta_{II}=3\beta \text{;}$ $k_{I}=\frac{1}{3}k,k_{II}=\frac{1}{3}k\text{;}$ $w_{I}=0.8w,w_{II}=0.8w$	11399	2092824
			$\beta_{I}=\frac{1}{3}\beta ,\beta_{II}=3\beta \text{;}$ $k_{I}=3k,k_{II}=\frac{1}{3}k\text{;}$ $w_{I}=1.2w,w_{II}=0.8w$	2979	557626
			$\beta_{I}=3\beta ,\beta_{II}=\frac{1}{3}\beta \text{;}$ $k_{I}=\frac{1}{3}k,k_{II}=3k\text{;}$ $w_{I}=0.8w,w_{II}=1.2w$	10476	2015584

## Data Availability

Data will be made available on request.
